# The Quest for Innovation: Addressing User Needs and Value Creation

**DOI:** 10.1007/978-3-030-50991-0_1

**Published:** 2020-10-08

**Authors:** Hugo Campos

**Affiliations:** International Potato Center, Lima, Peru; International Potato Center, Lima, Peru

**Keywords:** Business models, Design thinking, Jobs to be done, Behavioral change, Failure, User needs, Pain of the customer, Satisfaction gaps, Disruptive innovation, Nonprofit, Prototypes, Value proposition, Value creation, Value

## Abstract

In agricultural and agrifood systems, like in many other economic sectors, the main innovation drivers have traditionally been (1) technological advances and (2) research and development. When innovation fails to address the actual needs of clients and end-users, however, satisfaction gaps are created. The result is that investors receive insufficient returns and end-users receive less than expected value. The consequences of failure can be deeper than just financial, however. Successful innovation in agriculture and agrifood systems is critical to secure affordable, nutritious, and safe food for all people. Rapid innovation is needed to address the serious challenge of climate change and to reduce agriculture’s global environmental footprint. The overarching goal of agricultural innovation should be to deliver high value to end-users and improve their quality of life and well-being. To achieve this goal, organizations must first understand the jobs to be done concept for their end-users. They must take into account user satisfaction gaps and frustrations. Understanding user needs is *as important* as producing technology to finding innovative solutions. This is true around the globe; in industry and public sectors, and in both developed and developing countries. This chapter explains why *investing* in innovation is very different from *succeeding* at it. Ultimately, the examples, ideas, and guidelines in this chapter can be brought to bear on agricultural innovation efforts (and any other economic sector), to make them more productive and worthwhile for end-users and investors/funders. The information herein is meant to increase the likelihood of successful innovation efforts at both profit-seeking firms and nonprofit organizations.

## Why Innovation?

There is nothing more difficult to take in hand, more perilous to conduct, or more uncertain in its success, than to take the lead in the introduction of a new order of things. For the reformer has enemies in all those who profit by the old order, and only lukewarm defenders in all those who would profit by the new order, this lukewarmness arising partly from fear of their adversaries… and partly from the incredulity of mankind, who do not truly believe in anything new until they have had actual experience of it. – Niccolò Machiavelli

The need to innovate is more urgent than ever before. While every economic sector has this need, few are more pressing than agriculture and agrifood systems, as they provide, every single day, food, feedstuff and fibers to humankind. The unforeseen arrival, and far reaching impact, of the COVID-19 pandemic, only reinforces the pressing need to get much better and effective at innovation efforts.

There have been many successful innovations in the field of agriculture to date, leading to increases in farmer productivity and production in many developed and developing countries. As a consequence, the number of people employed as farmers has fallen dramatically in some parts of the world: in 1900, 4 in 10 US jobs were in agriculture, whereas in 2010 this number fell to 2 in 100 (Autor 2014). However, in Africa and Asia, agriculture still remains as a significant source of jobs and income for many people. In Africa alone, despite declining as a share of employment, self-employed farming remains the single largest source of employment, where it accounts for over 50% of all employment in most countries and 35–54% of full-time equivalent-based employment (Jayne et al. 2017). These farmers require to benefit from innovation to boost their chances at success, achieve higher productivities, reduce their workload and gender gaps, and feed more hungry people (Box 1.1). Often, however, new technology and/or ideas that are presented to farmers fail to live up to their initial promise.

### Box 1.1 Main Imperatives Driving the Need to Innovate in Agriculture and Food Systems in Developing Countries

There are more than 820 million people in the world who are hungry, an already daunting figure likely to increase because of COVID-19. Hunger is on the rise in nearly all sub-regions of Africa – nearly 260 undernourished people live in sub-Saharan Africa alone. Asia contains the largest undernourished population – over 500 million people. Nearly 15% of the people in Southern Asia are undernourished. More than 2 billion people, mainly in sub-Saharan Africa and Southern Asia, are micronutrient deficient.

Systems for producing, packaging, and delivering food are responsible for 20–30% of global greenhouse gas emissions, 70% of freshwater withdrawals, and 70% of biodiversity loss.

Twice as much water will be required to produce sufficient food in 2050, but nearly one-third of agricultural production today takes place in water-stressed regions.

Climate change will increasingly stretch current food systems.

Source:

http://www.fao.org/3/ca5162en/ca5162en.pdf, https://www.weforum.org/reports/innovation-with-a-purpose-the-role-of-technology-innovation-in-accelerating-food-systems-transformation [verified on October 25^th^, 2019]

There is a critical need to increase the rate of innovation success in agriculture and agrifood systems, to address the so-called *wicked problems* plaguing agriculture associated with climate change and sustainability, as well as to match expectations in terms of adoption and value creation. The goal of innovation in agriculture and agrifood systems is beyond simply achieving financial goals or market share targets. It is also more important even than reaching a target number of users. While these are valid, relevant objectives, they ought to be understood as means to an end: to deliver greater value by means of improved well-being and outlooks, and quality of life for millions of farming families and consumers around the world.

*What leads to innovation? Is it simply good technology?*

Over 20 years ago, Steve Jobs, former CEO of Apple, said: “One of the things I have always found is that you have got to start with the customer experience and work backward to the technology. You cannot start with the technology and then try to figure out where you are going to try and sell it” (JWWDC 1997). Technology is simply a driver of successful innovation. It operates within an ecosystem of individuals and nonprofit, government, and private organizations focused on the social and economic use of new products, processes, and organization forms. Technology alone will not produce enough nor sustained success in any sector, including agriculture. I would posit that, increasingly in today’s and tomorrow’s world, technology alone does not represent the main determinant of the perceived value innovations create for its users.

*Is the answer a big budget?. Do more money and larger teams mean more innovation?*

Large budgets are not necessarily a good predictor of innovation success. A myriad of well-funded innovation efforts has fallen short of expectations or outright failed in the market. Case in point: Motorola’s failed launch in the 1990s of Iridium– a mobile telephone service providing global coverage that cost investors over $5 billion. While Iridium was expected to capture millions of customers, by the time it filed for bankruptcy protection, it had only acquired about 55,000. It was finally purchased for the discounted price of $25 million. Many modest budgets, in contrast, have managed to deliver high value to both investors and users. See Box 1.2 for a cassava example.

*If it’s not technology or money, what leads to successful innovation?*

One pivotal part of the answer is *people*. People are the most effective predictor of success of any innovative undertaking. People cannot thrive, however, in organizations that stifle their creativity and experimentation. Organizations must support, encourage, and foster a culture that enables employee innovation, or they risk crippling the talent they hired to produce results in the first place, leading employees to look for a brighter outlook and professional development elsewhere. To succeed, organizations must invest in building a system that both nurtures human innovation and takes the user into account.

Most innovation failures stem from failing to recognize that innovation is both an economic and social process rather than simply a technical one. Many innovation endeavors fail despite funding availability, talented people, strong technology, and sincere intentions. Ultimately, innovation success depends on whether the value developers *think* they are delivering matches the *actual* value users^1^ find in the product or service. The business model associated with any innovation is also pivotal to fulfilling users’ expectations and determining if an organization survives.

*Why do we have to innovate? Isn’t maintaining business as usual good enough?*

The simple, straight, and honest answer is *no*. Failure to address disruptive change and implement business model innovations has led to the demise of many successful companies and organizations, in both developed and developing countries.^2^

The pace of change is increasing rapidly. While this shift can be cause for concern in traditional organizations, it also presents an opportunity – a bright outlook for those bold enough to launch innovations that contest the *status quo*.

Innovation is the key to how an organization not just survives but thrives instead.

### Box 1.2 Innovative Ways to Create Value Out of Agricultural Byproducts

Cassava is the main crop in Africa on a wet weight basis, with Nigeria being the world’s largest cassava-producing country^3^. Cassava peels make up 20% of the whole root, but are discarded during processing. The peels amount to nearly 40 million tons per year in Africa alone, giving cassava a bad name as an environmental polluter with the mountains of waste around processing centers. To create a business opportunity out of this undesirable byproduct, the International Institute of Tropical Agriculture has developed high-quality cassava peel (HQCP) feed ingredients from wet peels. This innovation enables rapid water removal and accelerates the elimination of hydrocyanide. The intermediate product (60% dry matter, up from 30% in fresh peels) is safe for livestock to consume and stable for up to a week and can be sun-dried or heat-toasted to a storable product (90% dry matter). This can be done any time of the year in a small- and medium-scale setup or flash-dried in a more industrial case (see http://bit.ly/2j7bRu3). Since three tons of fresh peels yield about one ton of HQCP, Africa’s cassava peel waste could generate at least 12 million tons of HQCP annually – equivalent in metabolizable energy (ME) to 8 million tons of maize thus spared for direct human consumption. In addition, there is willingness to pay for HQCP; for example, when maize prices reached $300, HQCP was being purchased for $150. This ratio holds for wide price bands.

The huge value creation of this high-impact innovation provides an alternative source of feedstuff, protects the environment, and provides new income sources to smallholders producing cassava. It has been supported by the CGIAR Research Program (CRP) on Root Tubers and Bananas (RTB), and it leverages the expertise of several private and public partners in Nigeria, such as the National Office for Technology Acquisition and Promotion (NOTAP), Raw Material Research and Development Council (RMRDC), Bank of Industry, and SingleSpark® from the Netherlands, makers of FeedCalculator®.

Source:

Dr. Iheanacho Okike, International Institute of Tropical Agriculture, (i.okike@cgiar.org)

## Toward a Working Definition of Innovation

Since more than a century ago, the seeds of these ideas were already present. In Austrian economist Joseph Alois Schumpeter’s book, *The Theory of Economic Development* (1911), the concept of “creative destruction” was articulated as a process “that incessantly revolutionizes the economic structure from within, incessantly destroying the old one, incessantly creating a new one.” He also described innovation as the cause of market dislocations, which enabled new firms to displace old firms from markets (Schumpeter 1942).

Schumpeter pioneered the idea that competition should not be based only on price, but instead on capabilities and performance. He was the first known economist to shift the basis of competition from the ability to reduce costs to the capability to innovate.

Building on these ideas, for the purposes of this book, we will use Scott Berkun’s definition of innovation. Berkun, author of *The Myths of Innovation* book *,* states that “innovation is significant, positive change.” Innovation is something you work toward. It should be the end result or outcome. It is the sum of an organization’s efforts to keep its value proposition to customers and end-users both relevant and compelling.

Though such definition of innovation appears to be unexpectedly unassuming and simple, be mindful that the articulation of all the components leading to the successful practice of innovation, and the ability to claim success in terms of significant value actually delivered to customers, is a remarkably complex, yet attainable, endeavor.

## The Deep Relationship Between Innovation, Design, and Human Needs

The growing awareness that technology is only one driver of successful innovation has led to the development of a more human-centered approach to innovation. This approach is strongly anchored in a deep understanding of customers and other stakeholder needs, preferences, non-spoken and poorly articulated perspectives, and emotions. Here the attention is not on focus groups or on collecting and analyzing a deluge of marketing data. Instead, human-centered innovation identifies the sources of *meaning* for users.

Because users have a rather limited ability to articulate their unmet needs, innovators increasingly borrow the perspectives of designers to capture such needs. This goes well beyond purely aesthetic aspects and instead builds on empathy, visualization, enlightened failure, and market experimentation. A design perspective aims to understand the behavior that people struggle with while living their lives, bringing a human-centered perspective to innovation efforts. Only through the willingness to immerse yourself in the lives of users can you uncover their constraints and frustrations with non-existent or limited solutions. That immersion simply cannot happen in your office or within the boundaries of your organization. Instead, you must empathize with the pain, disappointment, and frustration your users endure in their daily lives*.*

In the agricultural and agrifood sector, there have been diverse attempts to understand what the needs of farmers, members of supply chains, and users look like and to involve them in the technology design and innovation processes. However, a deliberate and explicit consideration of human-centered design to address how technology fits into the social, cultural, and emotional context of its users is not common practice. This is unfortunate, as such considerations would only increase the likelihood of success of innovation efforts, as well as the ability of innovation practitioners to truly apprehend how value is perceived by farmers and other participants in food systems. Fortunately, several diverse human-centered methods to innovate have been already developed in other domains. The following section briefly describes one approach – design thinking.

### A Primer on Design Thinking

One highly effective approach to create human-centered products and services is design thinking.^4^ It is typically structured as three overlapping phases:

- Inspiration
- Ideation
- Implementation

Typically, these phases proceed sequentially, although sometimes design thinking loops back onto itself as solutions are tried out. It is quite different from highly linear or more rigid approaches to innovation.

 Design thinking shapes the experience of both innovators and users. It recognizes organizations as human groups and factors in the importance of emotions. It encourages learning and engagement. By closely involving users in the identification of the opportunity and its solution, design thinking garners a wider commitment to change (Liedtka 2018). Among its stand-out main characteristics, design thinking is human-centered, possibility-driven, option-focused, and iterative. Design thinking starts with real human beings instead of demographic data, and before even attempting to generate solutions, it purposely delves into the lives, challenges, and circumstances of the people whose lives we aim to improve. It is possibility-driven since it addresses the question, “What if anything were possible?” and it focuses on developing multiple options before zooming in on the most appropriate one. Furthermore, design thinking is iterative, as it sails through several rounds of prototypes and multiple rounds of real-life experiments to refine ideas and potential solutions (Liedtka et al. 2017).

 Design thinking is a highly effective, pragmatic innovation and problem-solving approach.

#### Inspiration

During the inspiration phase, multidisciplinary teams look for user satisfaction gaps. These gaps are sometimes referred to as the “the pain of the customer.” They are different, however, from the “voice of the customer.” The most effective way to apprehend satisfaction gaps is to learn directly from the people you seek to help. Identifying these gaps cannot be achieved through surveys, interviews or focus groups, or demographic data, though. Instead, you must immerse yourself in the lives of users to deeply understand their needs, pains, and aspirations.

One option to achieve that, among many alternative ones, is to develop a detailed map of the journey users take in their quest to address their satisfaction gaps. An ethnographic analysis of all the contact points occurring between users and goods/services is also an effective approach. The construction of empathy maps and trajectory maps are also effective approaches during this stage. The focus of inspiration should not be on data collection and analysis, but rather on gaining insight about what makes for a meaningful customer journey (Liedtka 2018).

#### Ideation

 Ideation ensues inspiration, and it aims to detect unarticulated gaps between what users require and what they actually receive. This phase generates, develops, and tests entry points for design. Field observations and research are distilled into insights to discover solutions or drive change.

The inspiration and ideation phases are about divergent thinking, where on-purpose insights are made to clash against each other. Openness, empathy for others, sheer curiosity, and the ability to learn by doing are essential to this process. At this stage, the visual representation of concepts through early draft prototypes can help explain complex thoughts and help the team gain insight. During ideation, people identify themes, create insight statements, and attend co-creation workshops where users and innovation developers jointly work together to establish solutions.

#### Implementation

 Implementation is the ensuing phase, when the innovation solution actually gets developed. The best concepts from the ideation stage should point to a clear way forward through prototyping. Prototyping uncovers potential implementation challenges and unintended consequences of products or services.

 Prototypes anticipate how people understand the potential solutions to their satisfaction gaps. The team develops several iterations of concept prototypes. Later on, functional prototypes are tested, and a “looks like” design model is agreed upon. Prototyping plays an especially important role in developing countries where innovation ecosystems are generally weak or incomplete. Prototyping enables innovators to experiment, learn, and adapt concepts, so they can quickly and cheaply refine the product into something even better.

Successful design thinking does not just end with a product or service that simply reduces the pain of the user. The solution must then be communicated to a wider audience through powerful, compelling narratives and storytelling for wide product or service adoption to take place.

A word of warning – design thinking can create substantial tension in organizations and companies accustomed to linear, milestone-based product development and innovation. One of the tenets of design thinking and human-centered design is the need to achieve a close rapport with users – something at odds with a more structured view of developing new products and services.

Additionally, design thinking not only embraces but actively seeks failure as a key learning approach. Yet, many organizations have a near zero tolerance for failure. That means that the implementation of a human-centered approach in organizations requires strong leadership and full organizational support, or else it is likely to fail, once again leading to innovation efforts falling short of expectations (Bason and Austin 2019).

## Innovation Is Different from Research and Development

Often innovation and research and development are referred to as if they are interchangeable concepts. Nevertheless, their meaning, scope, and purpose are quite different: research and development (R&D) comprise creative and systematic work undertaken in order to increase knowledge – including knowledge of humankind, culture, and society – and to devise new applications of already available knowledge (OECD 2015). This differs in many ways from the working definition of innovation provided in Sect. 1.2.

The difference between the two can create misunderstandings, as well as conceptual, practical, and very expensive mistakes. Though in theory, corporate R&D spending relates to increased innovation and the growth of revenues and profits, that’s not always the case. The magnitude of R&D expenditures is only moderately related to the number of innovative products/services launched or even those that gain high market share and profits.

To some extent, confusing R&D and innovation arises from a narrow, technology-driven stance on innovation. Innovation, by default, encompasses a much wider scope than R&D, and as Gina O’Connor from Babson College aptly puts it, innovation involves three diverse capabilities: discovery, incubation, and acceleration. In many cases, the resources, investments, and time required during the incubation and acceleration stages far exceed those needed to develop technologies in the first place.

Indeed, within profit-seeking firms, R&D and market success are two different things. *Strategy &* – a business unit within PricewaterhouseCoopers – was unable to find a statistically significant relationship between R&D spending and sustained financial success when it analyzed the top 1,000 most innovative companies over 12 years. Spending on R&D was also found to be unrelated to growth in sales or profits. Moreover, the top 10 most innovative companies are rarely the top 10 spenders on R&D (Viki 2016).

*Which main aspects separate the most innovative companies from less innovative ones?*

More innovative companies show:

- Close alignment between innovation and business strategy
- Company-wide cultural support for innovation
- Close involvement by leadership with innovation programs
- Deep understanding of insights from end-users

This trend was again observed in 2018 (Table 1.1), when only two out of the five most innovative companies, namely, Amazon and Alphabet, also ranked among the five top R&D spenders.

**Table 1.1:** Top R&D spenders and innovative companies (http://www.strategyand.pwc.com/innovation1000 [verified on October 11^th^, 2019]) in 2018

Most innovative companies		Top R&D spenders	
Rank	Company	Rank	R&D spending (US $ billion)
1	Apple	1	Amazon (22.6)
2	Amazon	2	Alphabet (16.2)
3	Alphabet	3	Volkswagen (15.8)
4	Microsoft	4	Samsung (15.3)
5	Tesla	5	Intel (13.1)
6	Samsung	6	Microsoft (12.3)
7	Facebook	7	Apple (11.6)
8	General Electric	8	Roche (10.8)
9	Intel	9	Johnson & Johnson (10.6)
10	Netflix	10	Merck (10.2)

Analyzing R&D intensity – a metric defined as the ratio between R&D expenditure and total revenue – sheds light on the effectiveness of innovation efforts. Dramatic differences can be verified even among the most innovative companies. Although Amazon, Alphabet, Microsoft, and Tesla show a large R&D intensity, within a 12–15% range, in the case of the most innovative company of 2018 – Apple – its R&D intensity only reached 5%. Furthermore, in the case of Nokia, despite a high R&D intensity in 2018 (21%), it ranked lower than 20th in terms of innovation.^5^

It might be a little unfair to compare firms across diverse industries and R&D intensity landscapes. Regardless, such comparison sheds light on the actual nature of the relationship existing between R&D efforts and the ability to translate them into products and/or services that are actually adopted by users and therefore create value.

Markovitch et al. (2015) provide reinforcing evidence regarding the lack of a direct relationship between R&D efforts and innovation outcomes: Using data from 141 US firms across a decade of data, they could not find any statistically significant relationship between a firm’s investments in basic, exploratory R&D (measured by each firm’s number of patents over the previous decade, weighted by how scientifically novel they were) and the firm’s stock market value.

This should not be a surprise once the meaning and purpose of innovation are understood. R&D can only contribute to the development of goods or services that are technically superior to previous alternatives.

*What about other examples?*

As you will see throughout this book, successful innovation requires much more than technical advantage. If this were the sole factor driving success, the Betamax system developed by Sony would have become the dominant design and the commercial winner in the video cassette recording market in the 1980s. Instead, it rather quickly became obsolete.

A successful example of limited R&D expenses related to a successful innovative product is the Japanese company Nintendo, which developed the Wii home video game console from off-the-shelf components. It achieved remarkable commercial success with a technologically inferior product compared to those of competitors. And it did it in a market where Sony and Microsoft spent much more on cutting-edge gaming consoles.

Yet another well-documented example about the non-existent or weak relationship between R&D spending and innovation success is the garment industry in the Philippines. Despite no access to formal R&D or academic collaborations, it has introduced incremental innovations in both products and processes, through reverse engineering and combining knowledge on new ways to successfully innovate and remain competitive in global markets (Rosellon and Del Prado 2017).

This is not meant to discourage R&D efforts. Many successful innovations have required long and expensive public or private research efforts. Many companies have translated large R&D budgets into successful innovations that are rapidly adopted. The takeaway message here is that an expensive R&D effort does *not guarantee* innovation success. The opposite also holds true: it is perfectly feasible to achieve large returns from innovation and become a successful organization in the field of innovation with a rather limited R&D investment. Furthermore, Open Innovation (see Chap. 10.1007/978-3-030-50991-0_3) is a very effective approach to increase the ratio of success of innovation efforts.

Innovation can succeed regardless of an organization’s size, the nature of its users, or the economic sector within which it operates. That’s encouraging news for young start-ups, small, and cash-strapped organizations. If they have the talent and boldness to lead, design, and execute innovation plans, they have a high likelihood of successfully challenging and even defeating older, larger, and much better-funded organizations.

## Does Innovation Only Pertain to Profit-Seeking Organizations?

Most of the insight, experience, and literature relevant to innovation relates to for-profit efforts where companies and organizations innovate to remain in business. They grow and adapt to the needs and desires of customers and markets along the way. However, that does not mean that the natural domain of innovation is only within profit-seeking organizations. Leewis and Aarts provide an updated perspective of innovation adoption in agriculture in Chap. 10.1007/978-3-030-50991-0_4 of this book.

 Nonprofit organizations , regardless of their role, size or geographic footprint, are also under increasing pressure to innovate. Their users and beneficiaries have needs that continuously evolve. They must adapt to the needs and expectations of their donors and funding agencies, as well as to the increasing scrutiny from governments, international bodies, and philanthropic organizations.

From this perspective, this chapter argues *that nonprofit organizations already run a business*. They might not seek profits but, nonetheless, they remain a business (Box 1.3). Regretfully, even today, the word “business” raises concerns, and it is not taken well within some university and other knowledge-seeking organizations.

### Box 1.3 The Actual Meaning of the Word Business

Often the word “business” encounters skepticism, diverse opposition, and even a degree of disdain in academic and nonprofit organizations. This is unfortunate, as the actual meaning of this word has very little to do with corporate greed or utter disregard for anything other than a ruthless pursuit of profits.

The word “business” is derived from the old English word *bisignes*, “attend to, be concerned with, be diligent.” Its actual meaning is “moving towards a goal, to be determined about serious action.” We advocate the use of this word because it conveys a positive activity worth pursuing.

*Are there good examples of an innovative nonprofit?*

Several Boxes provided throughout this book describe successful nonprofit innovations. A further example of an innovative nonprofit model is the Innovation Lab set up by Oxfam.^6^ In a systematic way, it addresses core development challenges that have potential for market systems solutions. Market-based ideas are analyzed, and pilot projects are run to make the solutions better.

*BlocRice* is an innovative program launched by Oxfam in Cambodia aimed at increasing rice farmers’ leverage to secure fairer prices. It is based on digital contracts established among rice agricultural cooperatives, exporters in Cambodia, and buyers in the Netherlands. Yet another very good example of innovation in a nonprofit organization is Mercy Corp’s Social Venture Teams, which acts as an internal incubation and acceleration lab. Its purpose is to develop innovations able to be scalable into business in emerging markets. Chapter 10.1007/978-3-030-50991-0_6 by van der Velde et al. in this volume delves into several, additional successful examples of innovation within the nonprofit domain in developing countries.

The increasing relevance of innovation for nonprofit organizations is best reflected by emerging rankings that highlight the most innovative nonprofit organizations.^7^ For instance, the magazine *Fast Company* has added nonprofit organizations to the large number of sectors it ranks on innovation.

*Does this extend to the public sector?*

The need to innovate also reaches the public sector since government delivers, as a monopoly, services to millions of citizens who sustain it with taxes. The public sector represents the main economic force in many countries, particularly in emerging ones. As global standards of transparency, accountability, and value for money become more stringent, governments are also increasingly pressured to innovate.

 Public sector innovation in developing countries is even more pressing and relevant, since public sector expenditures represent a larger proportion of gross domestic products (GDP) in many such countries than in developed ones. Furthermore, innovation in the agriculture and agrifood domains is particularly critical in developing countries, as their economic footprint is much larger than in developed ones. For instance, agriculture, food, and related industries accounted for about 37% and 5% of total GDP in Ethiopia and the United States in 2017, respectively. In other words, properly executed innovation in agriculture and agrifood systems has much greater leverage, spillover power, and a larger ability to create additional value and new jobs in developing countries than in developed ones.

While many governments are forced to adapt to a maelstrom of change, progress is often ad hoc rather than reliable, reactive vs. deliberate, and sporadic instead of systemic. The public sector has not yet ensured that innovation leads to consistent and reliable options that can deliver better outcomes and greater impact.

OPSI,^8^ the OECD Observatory for Public Sector Innovation, provides tools, resources, and models for public sector innovators. These are important because public-service institutions need to learn to innovate and to pay more attention to social, technological, economic, and demographic shifts. It’s the only way to cater to the increasing needs of their progressively empowered citizens.

*What’s a good example of innovation in the public sector?*

There are some encouraging examples from the public sector, though. One such example is NESTA (https://www.nesta.org.uk/) in the United Kingdom,^9^ which represents a remarkably successful example of developing innovation in the public domain. In addition to developing creative economy and innovation policies, NESTA impact includes, but is not limited to, health, education, and the arts.

In today’s world, the need to innovate spans the entire continuum from private to public sector – profit-seeking and nonprofit organizations. At its core, innovation is a way of life, an attitude based on continuously challenging the *status quo*, rather than merely reacting to changes in customers, regulations, competitors, and markets.

## Disruptive Innovations

Perhaps one of the greatest misunderstandings about innovation comes from the concept of “disruptive innovation.” When it works, it can allow smaller, less-funded organizations to disrupt the market share of established, incumbent ones (Christensen et al. 2015). Although there are certainly large organizations able to drive smaller organizations out of business, when disruption occurs, it can allow a proverbial David to overtake Goliath.

Products or services in and of themselves do not represent disruptive innovations, however. The concept refers to the *trajectory* of a product or service along the value curve rather than about a discrete product or service. Disruptive innovations need not be breakthrough innovations. Instead, they consist of products and services that are simple, accessible, and affordable (Dillon 2020).

### How Do Disruptive Innovations Unfold?

**Fig. 1.1:**
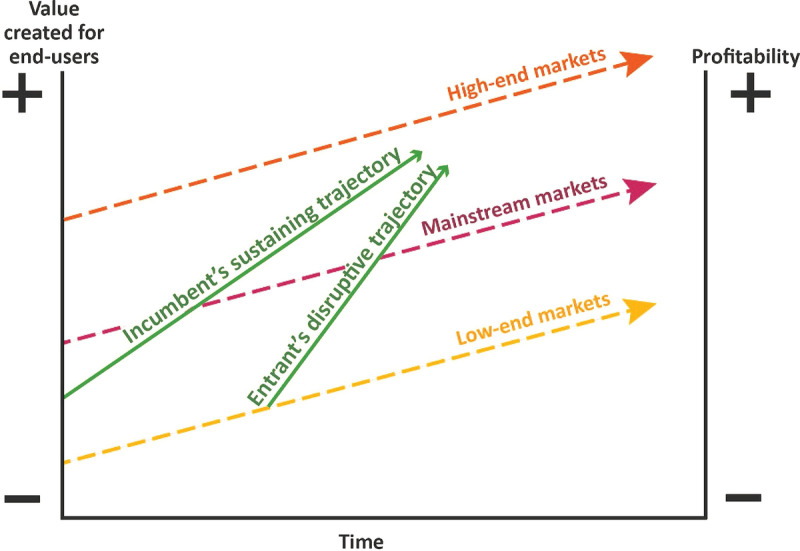
Trajectory in the market of disruptive innovations. (Modified from Christensen et al. 2015)

 Incumbent organizations usually focus on improving the value delivered to their most demanding customers. This follows the logic that these customers are the most profitable. The result is that the needs of only a fraction of users are met. Other customers are left inadequately served (incumbent’s sustaining trajectory in Fig. 1.1). These overlooked and/or non-consuming customers represent a significant opportunity to provide more than expected value at a (relatively) low price.

Since incumbents tend to focus on the most profitable, high end of the market, they usually fail to defend the least profitable segment as vigorously. This allows new entrants – the disrupters – to move up-market, improving value and quality while keeping the features that enabled them to challenge the incumbents (Entrant’s disruptive trajectory in Fig. 1.1). Innovations tapping into non-consumption are particularly relevant in developing countries, since they represent a means to alleviate poverty. They can open new markets, becoming the main building block toward greater prosperity, as argued by Christensen et al. (2017).

 Disruptive innovations originate either from the low end of markets or from tapping into products and services not yet consumed (Wunker and Farber 2019) (Box 1.4). One private sector example is low-cost air travel, a hugely successful disruptive innovation built upon addressing non-consumption (Taneja 2017). Many low-cost air carriers, such as Southwest in the United States, EasyJet and Ryanair in Europe, IndiGo in India, Azul in Brazil, Lion Air in Indonesia, and Sky Airline in Chile, have successfully challenged incumbent, larger airlines.

These no-frill air carrier fleets usually have only a single type of aircraft to reduce maintenance costs. They fly to smaller, secondary airports which are cheaper to operate.^10^ The cheap fleets reduce staff by issuing tickets and boarding passes digitally. They charge high fees for checking in with a staff person. This shift has enabled these companies to tap into a mass of consumers who would otherwise use cars, trains, or buses or just not travel at all. In other words, low-cost carriers do not necessarily compete against other airlines. Instead, they cater to the very large number of customers that would have not previously considered travelling by air.

In the case of low-cost air travel, the financial advantage of innovation came as a result of improving the quality of life of its users by making air travel affordable.

A relentless focus on the needs of users should also be aimed at by agricultural and agrifood systems innovations. If individual innovators address the unmet needs of users, returns on investment will increase.

In Africa, innovators who address the daily unmet needs of low-income consumers have a larger likelihood of success than if they chased after higher margin opportunities arising from the growth of the middle class. This market creation mindset is behind the shift from poverty to prosperity in Taiwan, South Korea, Singapore, and Hong Kong.

Another reason to focus efforts at the base of the pyramid, as discussed elsewhere in this volume by van der Velde and coauthors in Chap. 10.1007/978-3-030-50991-0_6, is that the actual growth of the African middle class has been much less robust than forecast (Christensen et al. 2017).

An example that illustrates how this principle works in action is the development of sweetpotato cultivars enriched in vitamin A, known as Orange-fleshed sweetpotato (OFSP) by the International Potato Center, which explicitly examined the unmet needs of end-users, many of whom were living in poverty. Such cultivars represent a rich, affordable source of beta-carotene, which the body converts into vitamin A (Low et al. 2017). Scientists at the International Potato Center challenged the conventional wisdom concerning food-based approaches and institutional barriers, to address the unmet need of having a diet with proper levels of vitamin A, particularly in children and lactating mothers. To date, a multi-partner, multi-donor initiative based on OFSP has already reached over 6 million households in sub-Saharan Africa, most of them representing the base of the pyramid.

#### Box 1.4 Tapping into New Markets: Rapid Diagnostic Tests for Malaria

Malaria is a severe public health issue. According to the World Health Organization (WHO), in 2016 there were over 215 million cases of malaria globally, which claimed nearly 450,000 deaths each year, out of which nearly 200 million cases and 400,000 casualties occurred in Africa. It remains as the single most deadly global health disease.

Blood tests are unaffordable for many Africans. Others are reluctant to go to hospital and be tested for malaria using traditional diagnostics. Instead, they rely on self-medication to treat potential cases. This creates yet another public health burden because many of the misdiagnosed cases are treated with antibiotics. This unneeded treatment accelerates the building up of antibiotic resistance, which has already rendered unsuccessful, previously highly effective drugs such as chloroquine and artemisinin.

The Baltimore, USA-based startup company Fyodor Biotechnologies saw an opportunity in this problem and developed a low-cost urine test for malaria. For less than $2 and without the need for access to health workers, this test provides an accurate diagnosis in less than 30 minutes. It is simpler to use than testing blood samples. It has already been launched in Kenya, and plans are in place for its commercialization in other markets.

Source: https://www.who.int/malaria/media/world-malaria-day-2018/en/ [verified on October 11th, 2019],

https://www.christenseninstitute.org/blog/innovators-creating-prosperity-fyodor-biotechnologies/ [verified on October 11^th^, 2019]

## The Dilemma of Exploration Versus Exploitation

One of the most intellectual and practical challenges that organizations face is how to balance the goods and services they currently deliver to customers, against the need to develop the future innovations their continued existence depends upon.

Many once-dominant companies have faltered when developing products needed to address market changes – to either retain current customers or gain new ones. Kodak excelled at analog photography and was the uncontested global leader for decades on end. It failed, however, to make the leap to digital cameras and adjust its business model to succeed in an increasingly digital world.

Organizations must create a *dual capacity* to exploit their current business while exploring new opportunities. These “ambidextrous organizations” have been extensively analyzed by Charles O’Reilly of Stanford University and Michael Tushman of Harvard University. Ambidextrous organizations pursue sustaining innovations that increase business success, instead of solely focusing on creating new markets. Typically, they have most of the traits shown in Table 1.2 and are able to pursue disruptive, exploratory innovations which are critical for the organization’s future (O’Reilly and Tushman 2004).

**Table 1.2:** Some traits of the exploitative and explorative domains observed in ambidextrous organizations

	Ambidextrous organizations	
	Exploitative business	Explorative business
*Type of innovation*	*Sustaining innovation*	*Disruptive innovation*
Strategic intent	Cost, profit	Learning, growth
Critical tasks	Operations, efficiency	Nimbleness, new business models
Culture	Efficiency, risk-averse, quality	Speed, risk-taking, flexibility
Leadership style	Authoritative, top-down	Visionary, involved

Adapted from Frederik (2015)

Much of the discussion on how to implement ambidexterity examines structural aspects, such as the creation of separate units to pursue new opportunities while keeping the same general manager to lead both the new unit and the parent company (O’Reilly and Tushman 2004). However, there are equally if not more relevant aspects to consider, such as having strong, shared values within the organization, which enables corporate headquarters to relinquish some autonomy to divisions without losing overall alignment and strategic fit.

Govindarajan (2016) recently developed a practical framework to allocate time and resources to the competing and opposing demands of managing today’s needs and tomorrow’s possibilities:

- *Manage the present core business at peak efficiency and profitability.*
- *Identify and abandon old and irrelevant practices, ideas, and attitudes.*
- *Convert breakthrough ideas into new products/services and business*.

Becoming proficient at both current business and future exploration is no small feat, though. It has only been achieved by a few companies. Recent work by Haan et al. suggests that no more than 2% of companies achieve it.^11^ Among the features shared by such a select group of companies, the following stand out: excellence at both exploration and exploitation; retaining an “outside-in” focus even when successful; embracing necessary disruptions – even if painful; and having in place a clear model for renewal.

## Innovation and Failure^12^

Most innovations launched to the market by companies fall short of revenue expectations. Approximately 80–85% of all fast-moving-consumer-good launches fail, according to 2018 research^13^ by Nielsen, the global measurement and data analytics firm. Though there is no equivalent data known on nonprofit organization innovations, it is unlikely that the opposite would apply to their goods and services.

This points to substantial shortcomings in the ability to match value propositions with the actual needs of customers and users. While the technology itself can certainly go wrong in the design process, the difficulty of designing the right business models – vs. simply the right technology – remains one of the most challenging components of innovation success.

- *How could it be that the vast majority of commercial and social innovations fail – falling short of their profit, adoption, and/or impact goals?*
- *How can we better articulate the social, emotional, and economic gains brought about by innovations so that their adoption rates improve?*
- *Why are people so reluctant to embrace change?*
- *Why do most organizations, both private and public, struggle in their innovation efforts?*

These are critical questions that every individual and organization genuinely interested in embracing innovation need to consider.

In the agricultural realm, the story of coffee illustrates how innovation can lead to tremendous success despite failure. Today, coffee^14^ is a regular part of the lives of people in most of the world. For many of us, it represents nothing less than a “must have” to start a productive day.

Yet, hundreds of years ago, coffee was banned, several times, in many parts of the world. In Mecca, it was banned because of the fear that coffeehouses could become sources of social agitation against religious authority. In Constantinople, during the reign of Sultan Suleyman, a fatwa against drinking coffee was issued in 1543. In Europe, the situation was not any different as coffee and/or coffeehouses were once banned or curtailed by King Charles II in England, King Frederick the Great in Prussia, and the Swedish Parliament!

Obviously, tremendous social and cultural changes have shaped our perceptions of coffee. Only two decades ago, coffee shops in the United States were generally small, locally owned places where people gathered over poor-quality, cheap coffee. Along came Starbucks, which changed the game by introducing Americans to high-end, European-style coffee drinks that are now a regular part of the lives for millions of people every day. How was Starbucks able to innovate so successfully in a relatively short period of time? A main driver has been its ability to adroitly cater to the needs of its customers and to the job they need to get done while drinking coffee. For instance, Starbucks does not just provide coffee for its customers. Instead, it provides a “third place” outside their homes and workplaces, which offers not only coffee but also a secure, warm, and welcoming environment where people can gather and connect.

As of 2018, Starbucks runs over 28,000 coffee shops and employs nearly 300,000 people worldwide. To put its global team size in context, that’s more people than the population of about 150 individual countries.^15^ Yet, Starbucks is having to innovate again. Starbucks Reserve is the coffee giant’s newest initiative of specialty outlets which are meant to compete with third-wave coffee shops that source unusual or small-lot beans and train baristas in hand-poured preparation methods. Ever-evolving customer needs have to be taken into account, or Starbucks may soon find itself going the way of Eastman Kodak.

The learnings from the development and adoption of a globally successful innovation such as Starbucks in the coffee sector can be widely applied to a wide range of sectors, regardless of the technology they use, the sector they work in, or the geography they serve.

The launch of new technologies represents social experiments. There are many psychological, social, and economic factors that individual and organizational innovators must understand. The sheer success and global adoption of Starbucks illustrate how success can be achieved if an organization takes into account the emotional and social underpinnings of its end-users.

The takeaway here is that tensions around innovation arise not so much from either the potential benefits or pitfalls an innovation represents. Rather, tension comes from hidden feelings and sentiments of insecurity, since innovations challenge seemingly stable economic interests and social institutions.

One positive way to frame innovation failure is: “failure is the experience that precedes success.” There is rarely successful innovation without a fair share of failure along the way. Every successful innovator deals with the frustration, pain, and anxiety associated with numerous setbacks. I actually invite you to embrace the concept of *clever failure*, namely to fail often, cheaply, and as early as possible in the innovation process. Because failure is inherent to innovation, organizations should embrace it and help employees brace for it, and provide mechanisms to de-risk it instead of shying away from it. Take pains to understand why consumers rarely adopt or purchase innovations at the rate developers expect. A basic understanding of behavioral economics provides insight and can help organizations plan for the way human beings act, adapt, and react.

### Loss Aversion

 Loss aversion refers to when losses loom larger than gains.^16^ This asymmetry between the power of positive and negative expectations or experiences has an evolutionary history. Species that treat threats as more urgent than opportunities have a better chance to survive and reproduce (Kahneman and Tversky 1979).

What has loss aversion to do with innovation failure? A lot. At its core, loss aversion is an expression of fear. Losses elicit stronger, more visceral feelings than comparable gains. When our customers, used to the value that a good or service provides, are faced with innovative alternatives, they might rather stick with the one they feel comfortable with to reduce the risk of losing the value they are used to receive.

This has obvious implications for the rate at which innovations are adopted and purchased. Agriculture provides excellent examples of how loss aversion can significantly obstruct the adoption of technologies. Ward and Singh (2014) elegantly demonstrated that more loss-averse farmers are less likely to switch to new rice cultivars, although the new cultivars clearly outperform older, popular varieties under both normal and drought conditions. No wonder that many new cultivars, across many different crops, fall short of expected adoption, and therefore do not achieve profit or impact goals.

### Status Quo ***Bias***

 Status quo bias refers to the tendency to stick to the current status of affairs. One implication of loss aversion is that individuals have a strong tendency to remain at the current *status quo*. This has been extensively demonstrated through decision-making experiments (Kahneman et al. 1991).

*Status quo* bias explains why we are biased toward the default option: Most people tend to use their default Internet browser instead of installing a new one which may provide more speed, improved security, and a better navigating experience. *Status quo* bias can make people stick to their usual choices even when not in their best interests. Recently, Karl et al. (2019) demonstrated that participants in health insurance programs in the US that provide different financial benefits could be classified from very low to very high *status quo* bias categories. A high *status quo* bias was associated with a higher rate of physical inactivity, a higher sum of unhealthy lifestyle factors, and a higher BMI.^17^

*What does it take to get end-users to adopt a new innovation?*

Innovative goods or services often require consumers to change their behavior. Organizations trying to innovate frequently fail to fully appreciate just how daunting that task is that they face. Consumers assess innovations in terms of what they gain and lose relative to existing products. John Gourville of Harvard University refers to this as the “curse of innovation.” Generally, consumers overvalue the existing benefits of an entrenched product, whereas innovators overvalue the benefits of their new products/services. The result is a large mismatch between what developers think users desire and what users really want. This widening gap between the two groups increases the likelihood of failure for even the most innovative new products.

Gourville (2006) suggests avoiding this mismatch by avoiding the launch of innovative goods or services that demand substantial behavioral change. He argues that organizations should target non-consumption and strive for benefits that outrun losses at least by a factor of 10 as a rule of thumb. A strong example of this principle is Google,^18^ and as a consequence, it was able to quickly displace other search engines when it launched its own. The principle also explains why many innovations touted as “the next big thing” quickly flop upon hitting the market. To claim success, an innovation must continuously gain market share, in either adoption or actual profit terms.

Within agriculture, the fast adoption of biotech crops in countries such as Argentina, Australia, Bolivia, Brazil, Canada, China, India, Mexico, Paraguay, South Africa, Uruguay, and the United States, among others, illustrates this concept as well: between 1996 and 2017, their global acreage grew over 100X, from 1.7 to nearly 190 million hectares, out of which 53% were planted in developing countries (ISAAA 2017). This fast adoption story relates to the ability of biotech crops to deliver features which strongly resonate with the pain farmers face in their daily lives at the farm, such as convenience, simplicity, flexibility, speed, and peace of mind in terms of controlling weeds and insects.

An additional way to reduce failure is to launch innovations that only require small or negligible behavior changes, a tactic successfully deployed by Toyota. Its hybrid electric vehicles (e.g., Prius) deliver a driving experience nearly identical to that of a gasoline-only car. Consumers didn’t need to make a major shift in their behavior to shift to this new product.

### Managing Failure

Above all, organizations need to resist doing nothing at all – never implementing new or creative ideas. Robert Sutton of Stanford University suggests that inaction is far worse than failure. Failure, after all, implies some sort of output. Because the quality of innovation is intrinsically linked to quantity of ideas, it makes sense to employ metrics based on quantity of ideas.

Examples of such metrics include how many prototypes built, patents filed, papers published, projects completed, etc. Without a high quantity of attempts, there can be no innovation. Therefore, output – regardless of success or failure – must be rewarded. This may seem unrealistic without additional information.

*So how do you reward failure?*

According to Sutton, organizations can do the following:

- Make sure people are aware that failure to execute new ideas is their greatest failure and that it will bring about consequences.
- Make certain everyone learns from past failures; do not repeatedly reward the same mistakes.
- If people show low failure rates, be suspicious. Perhaps they are not taking enough risks, or maybe they are hiding their mistakes, rather than allowing others in the organization to learn from them.
- Hire people who have had intelligent failures where lessons have been extracted that enabled subsequent success. Let others in the organization know that’s one reason they were hired.

In the pharmaceutical industry, although spending on research and development went up more than 300% industrywide during the 1990s, the number of new drugs approved by the US Food and Drug Administration (FDA) during that period dropped by 50%. Companies see drugs that have cost hundreds of millions of dollars in the R&D phase simply stall out or fail in the FDA approval process. If this attrition does not improve, drug development will become prohibitively expensive.

 Vertex Pharmaceuticals is one example of a company that on purpose tries ideas that fail in pursuit of solutions. Vertex uses biotechnology to create transformative medicines targeting diseases such as cystic fibrosis, Duchenne muscular dystrophy, and hemoglobinopathies, among others, and also pain. Paradoxically, Vertex has attempted to decrease the attrition rate by increasing it during the early stages of the innovation process. The driving idea is that the more ideas (molecular combinations) researchers can test, the better their chance of finding a few good ones. Consequently, many ideas lead to dead ends, but at a much earlier stage. Good leads – in this case, effective, safe drugs – have a better chance of getting approved and ultimately generating sales.

Vertex has purposefully placed itself at an intersection of disciplines and technologies where it generates thousands of new drug candidates every day. The goal is to produce as many potential drug compounds as possible to find the intersection of the few combinations that will provide Vertex with a breakthrough drug.

This is how it works in the Vertex example: supercomputers randomly combine molecules with different drug targets. They then throw out the combinations deemed to be ineffective. The remaining molecule combinations go to a team of computer scientists, biologists, chemists, medical doctors, manufacturers, and lawyers, who evaluate them and bring those with the highest potential to fruition. Some compounds are discarded quickly and others a bit later. Some get developed into drugs.

On any given day, Vertex’s computers generate thousands of combinations; the vast majority of these end up as failures. By increasing the output of ideas and being unafraid to fail often and quickly, Vertex also has a better chance of developing successful drugs. The strategy is working – at the time of writing, Vertex has already secured FDA approval for four of its products, and several more are in various phases of the approval process for human use.

## The Theory of Jobs to Be Done

One of the main reasons innovations fall short of adoption and value creation is the deep lack of understanding about what drives a user to choose one good or service over another. While marketing departments allocate talented people and significant budgets to reaching end-users, in many cases “the customer rarely buys what the company thinks it is selling him.”^19^

The jobs to be done (JTBD thereafter) theory^20^ addresses why people buy or decline to buy what companies try to sell them. One of this theory’s main advantages is that it dramatically increases the ability to predict the likelihood of innovation success, a welcome addition to the toolbox of innovators. It was developed by the late Clayton M. Christensen and colleagues at Harvard University.

At the core of JTBD is the tenet that users do not purchase goods or services. *Instead, people bring things into their lives to get a specific job done or achieve progress* toward a particular goal under specific circumstances. The goods and services that users acquire are really unconscious means to filling in satisfaction gaps. Unless we understand the “jobs” that people want to fill, the likelihood of innovation success is severely undermined *regardless* of budget size or innovator skill.

A “job” can only exist within a given context – the where, when, who, or what. However, aspects such as the user’s life-stage, family status, and financial status also need to be considered. These insights are powerful predictors of user behavior.

For instance, “I am short of money” is too vague to allow innovators to fully grasp the “why” at stake. When recast in the framework of a JTBD, however, innovators gain valuable clues needed to predict user behavior: “I am short of money to pay my mortgage which is a source of concern to me. I am a proud provider for my wife and young kids.” That kind of insight helps the innovation better meet the needs than the alternatives offered by competitors.

JTBDs can be quite different from solutions. For instance, many providers of MP3 reproducers focused on the presumed solution, namely, providing music. In contrast, Apple aimed the iPod at helping customers listen to music through a seamless experience. It reconsidered the whole business around personal music management, enabling customers to acquire, organize, listen to, and share music.

As value creation targets JTBDs, organizations and firms can not only improve what they already have but also target new, “blue ocean” markets.^21^

The deepest and most significant advantage of this framework is that it explicitly acknowledges that each JTBD consists of several dimensions (see Box 1.5).

### Box 1.5 The Anatomy of a Job to Be Done

To fully grasp the *why* behind purchasing decisions, you must understand the complex nature of people. Every effective job must account for all three of these components:

- *Functional* – it addresses practical, technical aspects.
- *Social* – it addresses behavioral aspects of users, as well as how the user interacts and relates to people and groups of people.
- *Emotional* – it addresses very deep, personal aspects of users such as feelings, sentiments, aspirations, and desires.

Think of the job to be done by a luxury Swiss watch:

- *Functional* component – it provides an exact, accurate, and reliable account of the passing of time.
- *Social* component – it conveys an aura of status and economic success, providing cues about social status and power. “I am one of the few who can afford this very expensive and exclusive watch; therefore, I am successful and powerful.”
- *Emotional* component – it represents, in this case, a deep sense of belonging to family, passing the watch on from generation to generation. It can also represent the deep act of love from a spouse or partner reflected in such an expensive gift.

Most innovation efforts are mainly driven by their functional components. That immediately reduces their likelihood of success, since it is the social and emotional components that are the ones representing a strong, if not the strongest, driver of user preferences and subsequent adoption when people are presented with many alternatives. Within the agricultural domain, innovations solely focused on productivity or technological progress, but which fail to account for cultural and social aspects,^22^ are also likely to fall short of expectations.

*What are some examples of successfully meeting or failing to address the JTBD?*

The failure of many innovations, including those on the cutting edge of technology, can invariably be traced back to their inability to address a JTBD for a large enough constituency. The Segway two-wheeled vehicle is a good example. This cutting-edge technology was introduced to great fanfare and included the strong financial support of very successful entrepreneurs such as Jeff Bezos of Amazon. Yet, it failed to receive widespread adoption. Its failure was due, at least in part, to not enough users truly *needing* it.

In contrast, understanding the *why* behind consumer buying decisions increases likelihood of success. Beat headphones have captured a substantial market share in the United States, even though they are a technically inferior product when compared to headphones produced by makers such as Bose or JBL. How can this be? Beat Electronics appeals to the deep craving for high status of most teenagers. In part due to the association with rapper Jay-Z, users of Beat headphones are seen as “the cool kids” in high school (Wunker and Farber 2019).

The effectiveness of addressing a yet-to-be articulated JTBD is evident in the exponential growth of the mobile phone application Snapchat. It reached 190 million daily active users in 2019 despite fierce competition. The customer base of Snapchat users is much younger than that of other social media. What is so unique about Snapchat? Its main differentiating feature is that messages only last for a few seconds on the recipient’s phone.

Why has Snapchat been so successful to date? The JTBD theory explains it: at the time of its launch, Snapchat successfully addressed the emotional component of its JTBD by addressing the needs of teenagers who have long avoided parental scrutiny.

More recently though, the innovations launched by Snapchat have fallen short of addressing the evolving user needs, and therefore its customer base has been eroded, with many of its users deciding to choose competing products such as Instagram.

Finally, a further advantage of the JTBD theory is that it enables organizations to better understand the competitive landscape, to gain insight about previously unnoticed competitors, and to understand the main drivers behind the *why* in the customer’s mindset, whether it be social or emotional.^23^

## Business Models

A business model represents how an organization creates, distributes, and captures value, and it is an oftentimes neglected aspect of many innovation efforts, which tend to focus on the good or service to be launched, instead of on how to manage the value created. In addition, as already mentioned, it is unfortunate that the business world tends to be associated only with profit-seeking endeavors. Even innovation teams with enough business acumen oftentimes spend insufficient time and resources on their business models.

The self-inflicted damage caused by organizations paying insufficient attention to designing the right business model not only reduces an innovation’s likelihood of success, but it also grants the upper hand to competitors.

*A business model is not a business plan.* Nor is it a projection of future cash flows, profits, and break-even points. A business model embodies how the organization views value creation, how its goods or services address the JTBD, and how it allocates its talent and resources.

The ability to design business models tests whether an organization is truly customer-centered. In many cases, organizations only pay lip service to keeping customer needs at the center of everything.

 Innovative organizations cannot operate without a business model. A well-crafted model lets the organization test hypotheses, formulate and address questions, and challenge underlying assumptions.

A business model can be a mighty source of innovation. Even for companies with a strong digital footprint, a well-designed business model is key to transformational growth (Johnson 2018). Business models can be nicely articulated with the *canvas* *framework* developed by Osterwalder and Pigneur (2010). A canvas is a visual chart which is very effective to describe the founding blocks of the business model of an organization, good, or service. Ulvenblad provides in Chap. 5 of this book a detailed account about business models in Sweden.

With the right adaptive business model in place, an organization can rapidly evolve and achieve success.

### The Components of Business Models

**Fig. 1.2:**
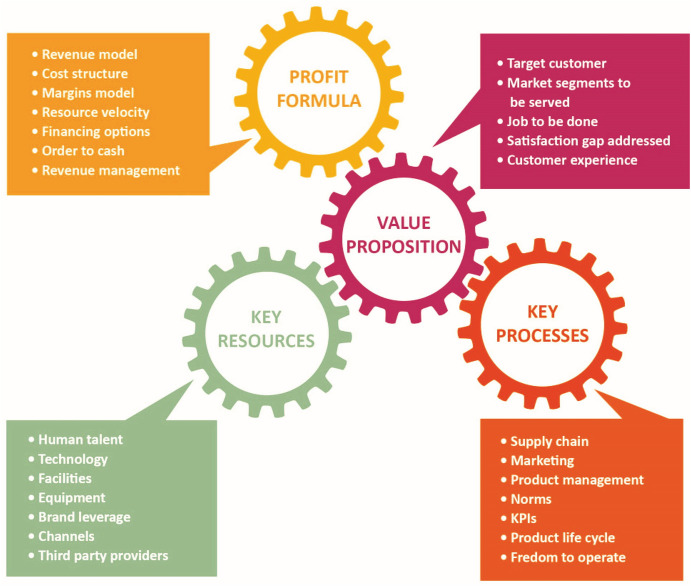
The four components of a business model. (Adapted from Johnson 2010)

A business model can be articulated around four components (Fig. 1.2):

Taken together, these four components can yield a powerful new way of doing business and achieving innovation.

#### The Value Proposition

 Value propositions are closely related to the JTBD concept. *The value proposition is the promise made to users.* A value proposition is a good or service enabling users to carry out a JTBD in a more convenient, effective, and/or affordable way (Eyting et al. 2011). It fulfills the job better than all other alternatives and at an appropriate price (Boxes 1.6 and 1.7).

While designing a value proposition, questions from the perspective of the customer – not from the organization – should be addressed:

- Is my JTBD properly and truly addressed?
- What will the good, the service, or a combination of them look like?
- Who is giving me this offer?
- How are they giving it to me?
- If it is a product or good, how do I dispose of it?
- What tradeoffs are imposed on me along with the offering?
- What does the landscape of payment options look like?

Once these questions are answered, a value proposition can be developed through experiments to better understand the JTBD.

##### Box 1.6 Hortifrut: Worldwide Leader in Berries Production

Hortifrut is a Chilean company and a global leader in the production and commercialization of blueberries. As the world’s second largest provider of berries, it enjoys a comfortable 25% of global market share of blueberries and has sales in over 35 countries. Its value proposition can be summarized as “berries for the world – every day.” That appeals to both wholesale customers and retailers such as Carrefour, Walmart, and Costco, as it simplifies and strengthens supply chains. At the same time, this value proposition enables the company to capture attractive prices all year instead of facing periods when prices drop because of excessive supply.

##### Box 1.7 Safaricom and M-Pesa: The Power of an Effective Value Proposition

While experimenting with diverse business models to develop a micro-loan business in Kenya, the telecom firm Safaricom found a much more compelling value proposition: a cheap, safe, and reliable system to send money by mobile phone to friends and family in rural areas. This insight became the key value proposition of M-Pesa (M for Mobile, Pesa for money in Swahili). M-Pesa is a branchless banking system that allows customers to pay bills, deposit, withdraw, transfer, and save money in a virtual account. In some markets, like Kenya, users can use a mobile phone to transfer money between the service and a bank account. As of 2018, Safaricom reported more than 28 million M-Pesa users and over 155,000 M-Pesa agents in Kenya. There are over 10 million active users in countries other than Kenya.

From its humble beginnings in 2007, M-Pesa has become a huge economic player in Kenya: it processes over 1.7 billion transactions a year, accounting for more than 50% of Kenya’s GDP value.

Ultimately, developing the value proposition is by far the most important component of any business model. Any organization wanting to innovate and succeed must spend time on developing the right value proposition before moving forward.

#### Profit Formula

The profit formula articulates how an organization creates value for itself, and for its shareholders/stakeholders. Its main components are (1) the revenue model, (2) the cost structure, (3) the margins model, and (4) resource velocity (Johnson et al. 2008). Revenue models establish the price at a given volume of transactions to cover overhead, fixed costs, and desired profit margins. Profit formulas also establish the resource velocity at which the organization turns over assets to achieve adequate returns. Profit formulas need to account for the customer’s willingness to pay.

Do not assume that business models and profit formulas are interchangeable. The profit formula is *an element of* a business model.

The value proposition defines value for the customer, whereas the profit formula establishes value for the organization and its shareholders/stakeholders (Johnson 2010).

#### Key Resources

 Key resources are competencies such as human talent, technology, facilities, equipment, channels, reputation, freedom to operate, and brand. They deliver in an effective way the value proposition to end-users. Key elements create value for the user and the organization. An organization needs to understand how those elements interact with each other. Generic elements found in every organization and which do not create competitive differentiation are not considered key resources (Johnson et al. 2008). For retailers such as Amazon, a key resource is its Information Technology infrastructure. Within the agricultural domain, examples of key resources can include the scientific expertise, market understanding, and proprietary knowledge at companies such as Bayer, Benson Hill, Calyxt, Cargill, Corteva, Enko, HZPC, Inari, Indigo, John Deere, KWS, Mosaic, Pairwise, Provivi and Syngenta among others, as their competitors may lack such resources. By leveraging such key resources, these companies increase the value and benefits their users get from the goods and services they offer.

#### Key Processes

 Key processes may include training, budgeting, manufacturing, planning, sales, and services. Such processes enable companies to deliver value propositions to users on a recurrent basis which is then able to be scaled up (Johnson 2010). In the case of Hortifrut, a key process is its ability to supply high-quality, fresh berries to customer throughout the whole year because it sources from production fields in different environments, including Chile, Peru, Spain, and Mexico. Building on previous expertise and local partners, it is also producing and commercializing berries in China. In the case of Tuskys, one of the leading supermarket chains in Kenya, which commercializes bread and pastries enriched with vitamin A, a key process is the method of incorporating the puree of vitamin A-enriched sweetpotato into their proprietary bakery operations.

## Closing Remarks

This chapter lays the foundation for successful innovation. These concepts can be applied to any economic sector – to profit-seeking and nonprofit organizations, and to both private and public endeavors. If there is one economic sector with an unparalleled application potential, however, it is agriculture and agrifood systems in both developed and developing countries. The ultimate goal of all innovation endeavors in this sector should be improving the quality of life, the outlook, and the well-being of farmers and end-users.

Firms seeking profits and nonprofit organizations remain as absolutely valid innovation pursuits. But such pursuits should be seen as a means to that end. Every innovation effort must allocate as much human talent and as many resources in developing business models as they spend on developing solutions which address the JTBD for end-users.

Taken together, the insights, concepts, and examples provided in this chapter articulate the pathway and the way forward to increased success and deeper impact of innovation efforts, which are urgently required in agriculture and agrifood systems.

## Footnotes

1 For the sake of simplicity, throughout this chapter, the term “users” will also encompass other parties toward whom innovation efforts are targeted, such as customers, clients, adopters, end-users, and beneficiaries.

2 The longevity of corporations is rapidly declining in the United States. In order to survive, they must innovate more rapidly than ever before. The 33-year average tenure of companies on the S&P 500 index in 1964 shrank to just 24 years by 2016 and is forecast to drop to just 12 years by 2027. Even large companies such as Alcoa, DuPont, and Yahoo all left the S&P 500 index in the period 2013–2017. Companies like these have been replaced by firms like Facebook, Under Armor, and PayPal. While the chance of remaining in the S&P 500 index for the first 5 years after being listed before 1970 was over 90%, companies listed from 2000 to 2009 only had a 60% chance of maintaining their grip on that rank (Anthony et al. 2018). A similar trend pervades all markets, including agriculture, both in developed and in emerging economies.

3 http://www.fao.org/faostat/en/#home [verified on October 11^th^, 2019].

4 A detailed discussion about design thinking goes beyond the scope of this chapter. Books such as *Change by Design* by Tim Cook and *Creative Confidence: Unleashing the Creative Potential Within Us All* by Tom Kelley and David Kelley provide a more extended perspective on design thinking.

5 https://www.strategyzer.com/blog/innovation-vefrsus-rd-spending [verified on October 11^th^, 2019].

6 https://www.oxfamamerica.org/explore/research-publications/oxfam-innovation-lab/ [verified on October 11^th^, 2019].

7 https://www.fastcompany.com/most-innovative-companies/2018/sectors/not-for-profit [verified on October 11^th^, 2019].

8 https://oecd-opsi.org/toolkit-navigator [verified on October 11^th^, 2019].

9 NESTA was formerly the National Endowment for Science, Technology and the Arts, which evolved into an independent organization in 2012.

10 In the case of London, Luton airport offers much cheaper fees to air carriers than flagship airports such as Gatwick and Heathrow. In the state of California, Oakland airport offers cheaper landing and taxiing fees than its neighbor, San Francisco airport.

11 https://bcghendersoninstitute.com/the-2-company-b9e5dc587b2b [verified on October 11^th^, 2019].

12 Throughout this chapter, we will refer to failure as the inability to reach expected sales, profit targets, adoption, or market penetration of innovations launched to the market. Though it is a rather narrow definition, it is a pragmatic, results-driven one and consistent with the book #x2019;s views on innovation.

13 https://www.nielsen.com/wp-content/uploads/sites/3/2019/04/setting-the-record-straight-common-causes-of-innovation-failure-1.pdf [verified on October 11^th^, 2019].

14 The late Calestous Juma, formerly of Harvard University, discusses the history of coffee in his book *Innovation and its Enemies* (2017), shedding light on the tensions and complex interactions arising among innovations, preexisting products, cultural landscapes, and social fabrics.

15 http://worldpopulationreview.com/countries/ [verified on October 11^th^, 2019].

16 Richard Kahneman, 2002 Nobel Memorial Prize Winner in Economic Sciences.

17 BMI (body mass index) is a person’s weight in kilograms divided by the square of height in meters. A high BMI can be an indicator of high body fat, although not always, and can be used to screen for weight categories that may lead to health issues.

18 When launched in 1998, the web browser Google provided its users with much more accurate results than existing search engines such as Altavista and Ask and faster results than Yahoo.

19 A quote from the late author Peter Drucker, known for his work on business management.

20 Developed and popularized by Clayton Christensen and colleagues at Harvard University, and by Anthony Ulwick from the consulting firm Strategyn.

21 Describing the concept of blue ocean markets is beyond the scope of this chapter. Interested readers are kindly invited to read the book *Blue Ocean Strategy: How to Create Uncontested Market Space and Make the Competition Irrelevant* by W. Chan Kim and Renee A. Mauborgne.

22 These can include gender norms or dietary preferences, depending on the market and country being served.

23 Amazon does not only compete with other providers of entertainment sharing the same technological means, namely streaming, such as Netflix or HBO. It also competes with any other unrelated provider of leisure and pleasure in the privacy of your own home.
